# Intracerebral Hemorrhage With Superimposed Psychiatric Illness: Navigating the Evaluation of Altered Mental Status

**DOI:** 10.7759/cureus.99704

**Published:** 2025-12-20

**Authors:** Risit Datta, Konstantinos Mouskas, Daniel Newman, Dylan Miller, Cristina Suarez Chiriboga, Steven Smith, Roxana Lazarescu

**Affiliations:** 1 Internal Medicine, Touro College of Osteopathic Medicine, New York, USA; 2 Neurology, Wyckoff Heights Medical Center, New York, USA; 3 Internal Medicine, Wyckoff Heights Medical Center, New York, USA

**Keywords:** altered mental status evaluation, bipolar disorder (bpd), intracerebral hemorrhage, multidisciplinary care (mdc), schizophrenia

## Abstract

Evaluating altered mental status (AMS) in patients with psychiatric illness presents a major diagnostic challenge, particularly when acute neurologic pathology such as intracerebral hemorrhage (ICH) is present. This report describes a 70-year-old woman with schizophrenia and bipolar disorder on haloperidol, lithium, and clonazepam who presented with confusion, rigidity, and agitation. Workup revealed a supratherapeutic lithium level and a right temporo-occipital intraparenchymal hemorrhage. The differential included lithium toxicity, catatonia, seizure, and hemorrhagic progression.

Multidisciplinary evaluation with neurology, psychiatry, and neurosurgery, along with EEG and serial imaging, identified right temporal epileptiform activity and stable hemorrhage. Gradual improvement followed medication adjustments and antiepileptic therapy.

This case underscores the diagnostic complexity of AMS in patients with coexisting psychiatric and neurologic disease. Overlapping symptoms can obscure acute intracerebral pathology, emphasizing the need for interdisciplinary collaboration, serial neuroimaging, and prompt EEG when indicated.

## Introduction

Altered mental status (AMS) encompasses disturbances in consciousness, cognition, or behavior and is a frequent presentation in neurological emergencies such as intracerebral hemorrhage (ICH). Diagnostic evaluation requires timely imaging, laboratory testing, and neurologic assessment to exclude life-threatening intracerebral pathology. However, in patients with chronic psychiatric illness, baseline abnormalities in cognition, affect, or responsiveness complicate the identification of new neurologic deficits. Differentiating psychiatric decompensation, medication toxicity, or catatonia from acute neurologic injury is often difficult.

The American College of Radiology (ACR) appropriateness criteria recommend expedited neuroimaging for adults presenting with new or unexplained AMS, delirium, or psychosis to exclude intracerebral processes requiring intervention [1]. In ICH, neuroimaging markers such as white matter disease burden correlate with delirium risk, further highlighting structural contributions to AMS [2]. In addition, catatonia and delirium frequently overlap in presentation, with features such as mutism, rigidity, and altered awareness, leading to misdiagnosis when psychiatric causes are presumed without adequate neurologic evaluation.

This case presents an elderly patient with ICH superimposed on chronic schizophrenia and bipolar disorder, whose evolving AMS necessitated serial imaging, EEG, and interdisciplinary management. It emphasizes diagnostic pitfalls and offers practical guidance for clinicians evaluating AMS in psychiatric patients with potential intracerebral pathology.

## Case presentation

A 70-year-old woman with a 20-year history of schizophrenia and bipolar disorder presented with worsening confusion, agitation, and rigidity over several weeks of steady progression in symptoms. Her husband reported increasing social withdrawal, incoherent speech, emotional lability, and disorganized behavior at home. She had been adherent to her prescribed psychiatric regimen, which included haloperidol (5 mg BID), lithium carbonate (450 mg daily), clonazepam (2 mg BID), benztropine (0.5 mg BID), and zolpidem (10 mg nightly). There was no recent history of trauma, infection, or medication adjustment.

On initial evaluation in the emergency department, the patient appeared disoriented, intermittently unresponsive, and minimally verbal, alternating between agitation and stupor. Her vital signs were stable and afebrile. Neurological examination revealed generalized rigidity, flat affect, and occasional purposeless movements, but no focal weakness or cranial nerve deficits. Given her psychiatric history, the initial differential diagnosis included catatonia, lithium toxicity, neuroleptic malignant syndrome, sedative effect, or worsening psychosis.

Laboratory studies demonstrated a supratherapeutic lithium level of 1.34 mmol/L (reference: 0.6-1.2 mmol/L). Complete blood count, basic metabolic panel, thyroid-stimulating hormone, and ammonia levels were unremarkable. Despite mild lithium elevation, the severity of her cognitive and motor impairment prompted further evaluation for neurologic causes.

**Figure 1:**
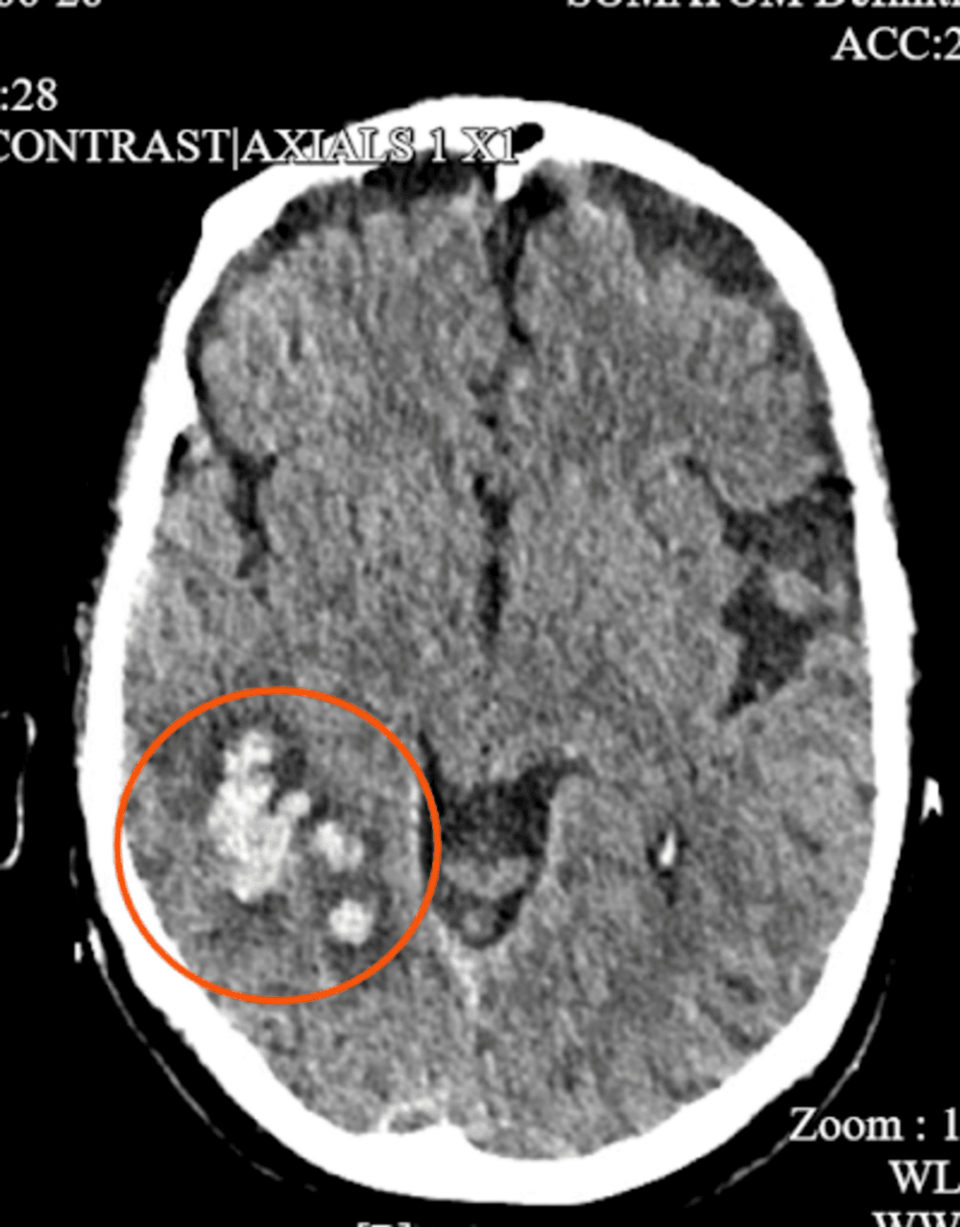
CT Head without contrast demonstrates an acute ICH at the posterior right temporal lobe, partially extending into the parietal lobe, with adjacent edema, measuring approximately 2.27 cm in width CT: Computed tomography scan; ICH: Intracerebral hemorrhage

A non-contrast CT scan of the head revealed a right temporo-occipital intraparenchymal hemorrhage with surrounding vasogenic edema. There was no midline shift or mass effect (Figure 1).

The patient was admitted to the intensive care unit (ICU) for close neurological monitoring. Neurosurgery recommended conservative management without surgical intervention. Dexamethasone (4 mg every 6 hours) was initiated to reduce cerebral edema, and levetiracetam (Keppra, 500 mg BID) was started for seizure prophylaxis.

Given the overlapping psychiatric and neurologic findings, psychiatry, neurology, and neurosurgery were consulted simultaneously. Psychiatry initially considered catatonia secondary to haloperidol exposure or lithium toxicity, but deferred lorazepam challenge due to concern for sedation. Neurology emphasized that lithium toxicity alone could not explain the patient’s profound mutism and rigidity in the context of an acute ICH, suggesting multifactorial encephalopathy.

Over the next 48 hours, the patient’s mental status fluctuated between lethargy, mutism, and periods of restlessness. She occasionally followed simple commands but remained largely nonverbal. Repeat laboratory studies showed normalization of lithium levels after withholding the drug (0.65 mmol/L), yet no corresponding improvement in cognition was observed. Given this discrepancy, nonconvulsive seizures or evolving encephalopathy were suspected.

**Figure 2:**
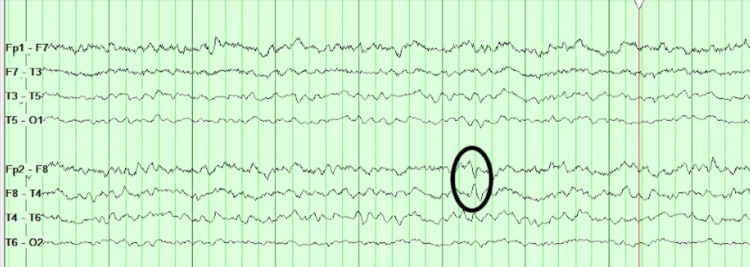
EEG demonstrates F8 discharge (epileptogenicity) on bipolar montage with some overriding right temporal slowing and dysfunction. EEG: Electroencephalography; F8: Lead electrode placed over the right anterior temporal/frontal area

An EEG was performed, revealing right temporal interictal epileptiform discharges with encephalopathy, rather than ictal activity, consistent with focal dysfunction adjacent to the hemorrhagic site (Figure 2).

**Figure 3:**
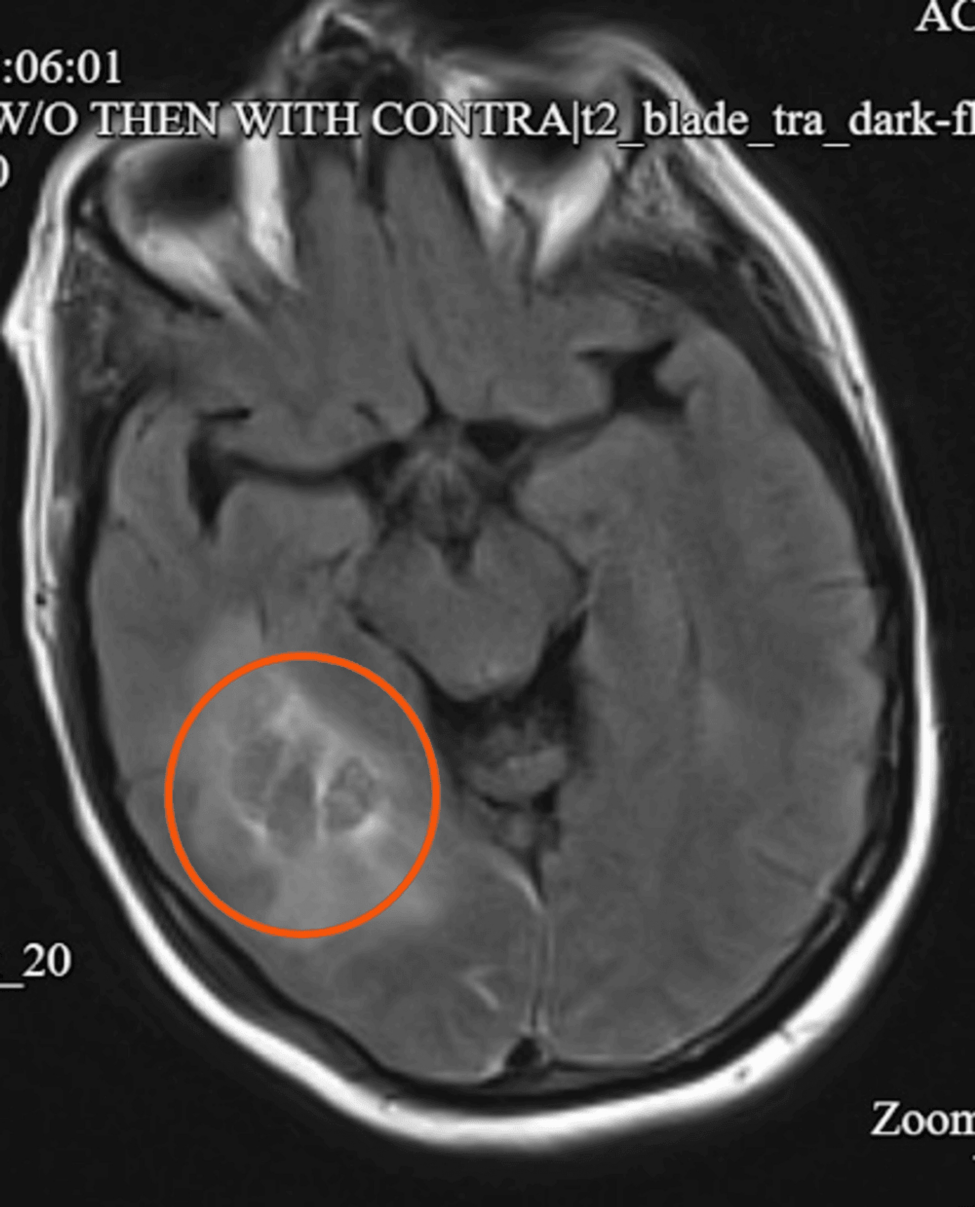
FLAIR MRI demonstrates subacute intraparenchymal hemorrhage in the right temporal lobe with significant surrounding vasogenic edema FLAIR: Fluid-Attenuated Inversion Recovery; MRI: Magnetic Resonance Imaging

MRI of the brain confirmed a stable right temporo-occipital hematoma with surrounding vasogenic edema and no underlying vascular malformation or neoplasm (Figure 3). 

Right temporal epileptiform activity corresponded to the site of the hematoma, indicating both seizure vulnerability and a likely contribution to her altered mental status.

Medications with sedating or extrapyramidal potential (haloperidol, clonazepam, benztropine, lithium) were systematically discontinued to reduce confounding factors and optimize neurologic evaluation. As her mental status remained variable, levetiracetam was transitioned to oxcarbazepine (Trileptal, 300 mg BID) due to somnolence from levetiracetam use. Daily neurologic checks and serial CT scans demonstrated stable hematoma size and resolution of edema.

During the following week, her mental status gradually improved. On hospital day 6, she began responding verbally and engaging with staff, though episodes of tearfulness and confusion persisted. Psychiatry cautiously reintroduced low-dose quetiapine (25 mg nightly) to manage mood instability and agitation while minimizing cognitive impairment risk.

By day 10, the patient was alert and oriented to person and place and able to engage in conversation. Neuroimaging remained stable, and her rigidity and agitation had resolved. Residual deficits included mild cognitive slowing and fatigue. At the time of discharge, the patient had stabilized neurologically and psychiatrically. She was transferred to a skilled nursing facility for rehabilitation, continued seizure prophylaxis with oxcarbazepine, and outpatient neurology and psychiatry follow-up.

## Discussion

Patients with chronic psychiatric illness pose significant diagnostic challenges when presenting with AMS. Overlapping neuropsychiatric features such as catatonia, delirium, and postictal states can mimic or obscure neurological deterioration. In the presented case, the differential diagnosis included psychiatric relapse, medication toxicity, ICH progression, and seizure activity. Resolving these required an iterative, multidisciplinary approach integrating imaging, EEG, and careful medication review.

First, distinguishing catatonia or delirium from neurologic causes requires systematic evaluation. Neuroimaging remains essential when AMS presents with an abrupt onset, new focal deficits, or fluctuating consciousness [1,2]. This aligns with ACR and AHA/ASA guidelines advocating urgent CT in unexplained AMS and repeat imaging when clinical status changes [1,3,4]. Both syndromes share motor and cognitive abnormalities, and misdiagnosis can delay treatment for the underlying brain injury.

Second, cortical ICH, particularly in temporal or occipital regions, predisposes to acute symptomatic seizures and nonconvulsive status epilepticus, which may manifest solely as AMS. Altered level of consciousness or AMS occurs in about 50% of ICH cases, meaning patients frequently present with global neurologic dysfunction even if focal deficits are absent or subtle [5]. EEG is indispensable when AMS fluctuates or lacks a clear etiology. Recent studies highlight EEG’s value in detecting nonconvulsive seizures after stroke and ICH [6-8]. In this patient, a continuous 24-hour EEG was performed to rule out nonconvulsive seizures, and the EEG localized epileptiform discharges to the hemorrhagic region, directing targeted antiepileptic therapy.

Third, medication review is crucial. Lithium toxicity, antipsychotic-induced catatonia, benzodiazepine withdrawal, and anticholinergic effects can each mimic or worsen neurologic dysfunction [8]. Temporarily withholding psychotropics with sedating or extrapyramidal potential clarified the patient’s neurological baseline and improved diagnostic accuracy.

Finally, structured neurochecks, serial imaging, and interdisciplinary collaboration were key in differentiating multifactorial encephalopathy from psychiatric relapse. Current literature supports ongoing reassessment, as overlapping neuropsychiatric syndromes, such as catatonia, may evolve dynamically [9-13].

While this approach is consistent with existing guidance, high-quality evidence defining standardized diagnostic algorithms for AMS in psychiatric patients with acute neurologic disease remains limited. Future studies should evaluate structured diagnostic pathways incorporating early imaging, EEG, and medication stratification to improve outcomes.

## Conclusions

This case highlights the complexity of AMS evaluation in patients with psychiatric comorbidity and ICH, displaying how overlapping clinical syndromes, medication effects, seizure activity, and structural brain injury can coexist and evolve. A structured, tiered diagnostic pathway including early imaging, prompt EEG when indicated, serial neurologic monitoring, and coordinated multidisciplinary care can help distinguish primary psychiatric relapse from life-threatening neurologic illness and guide safer, more effective management.
